# P13 A case of Kikuchi-Fujimoto disease triggered by an episode of bacterial cellulitis, associated with features of SLE

**DOI:** 10.1093/rap/rkac067.013

**Published:** 2022-09-28

**Authors:** Thushyanthan Guruparan, Sithu Bala, Jonathan Woodward, Benjamin Bennett, Rapti Mediwake

**Affiliations:** Department of Medicine, Barnet Hospital, Wellhouse Lane, Barnet, EN5 3DJ, London, United Kingdom; Department of Medicine, Barnet Hospital, Wellhouse Lane, Barnet, EN5 3DJ, London, United Kingdom; Department of Medicine, Barnet Hospital, Wellhouse Lane, Barnet, EN5 3DJ, London, United Kingdom; Department of Medicine, Barnet Hospital, Wellhouse Lane, Barnet, EN5 3DJ, London, United Kingdom; Department of Rheumatology, Barnet Hospital, Wellhouse Lane, Barnet, EN5 3DJ, London, United Kingdom

## Abstract

**Introduction/Background:**

We present a rare case of Kikuchi-Fujimoto disease (KFD), involving a patient journey from diagnosis, initial treatment, serological development and maintenance therapy. This is a disease of necrotizing lymphadenitis, often associated with autoimmune diseases such as Systemic Lupus Erythematosus (SLE) and antecedent viral infections. It has a higher prevalence in Asiatic people, affecting adults younger than 40 years and with a female:male ratio close to 1:1. It is a systemic disease with fever, cervical lymphadenopathy, rash and arthralgia being the most observed symptoms. Differential diagnoses include lymphoma, SLE and tuberculous lymphadenitis, mandating biopsy, although histological identification can be challenging.

**Description/Method:**

A 56-year-old male presented with pyrexia (38.7 °C) and bilateral leg swelling (left more than right), without evidence of heart failure or nephrotic syndrome. There was no other medical or drug history. His leukocyte count (13.4x109/L) and CRP (308mg/L) were elevated. Following intravenous antibiotics for leg cellulitis, his pyrexia and inflammatory markers improved. Subsequently, two weeks from initial presentation, he developed pyrexia (38-40 °C), sweats, weight loss and a rise in CRP (191mg/L) with a new pancytopenia (haemoglobin 107g/L; leukocytes 1.0x109/L; platelets 78x109/L) despite multiple courses of antibiotics, requiring intensive care support. Computerised tomography (CT) demonstrated axillary and para-aortic lymphadenopathy without evidence of infection. Blood culture, urine culture and serological infection screens were negative. His creatinine kinase (1573U/L), alanine aminotransferase (90IU/L), triglycerides (3.8mmol/L) and ferritin (14,000ug/L) were all elevated. Double-stranded DNA (dsDNA) antibodies, lupus anticoagulant and myositis immunoblot were all negative at this point, with normal C3/4. Anti-nuclear antibodies (ANA) (1:640) and anti-Ro/La antibodies were present. During this acute phase, there were no features of SLE; particularly, no mucocutaneous symptoms, serositis, arthritis or renal involvement. Bone-marrow trephine biopsy, performed to investigate pancytopenia, looking for evidence of macrophage activation, demonstrated no haemophagocytosis, but an elevated myeloid:erythroid ratio and bilineage dysplasia without abnormal lymphocytes. Lymph-node biopsy showed large areas of necrosis with karyorrhectic debris, histiocytes and lymphocytes; granulocytes were sparse with no granulomas, Reed-Sternberg or multinucleated cells. Immunohistochemistry revealed multiple CD68+ histiocytes, many also MPO+. Acid-fast bacilli were not seen. He was treated for necrotising lymphadenitis with three days of 500mg intravenous methylprednisolone and subsequently oral prednisolone, starting at 60mg daily, with clinical and biochemical improvement. During five-year follow up, he developed dsDNA antibodies, low complement and anaemia, all treated successfully with hydroxychloroquine. He did not meet the diagnostic criteria for SLE.

**Discussion/Results:**

The key to diagnosing KFD is histology from a lymph node biopsy. This typically shows necrotising lymphadenopathy. Without this, several differential diagnoses, including infection-related lymphadenitis, macrophage activation syndrome, adult onset Stills disease, SLE and lymphoma, should be considered. Thus, a prompt biopsy is crucial for diagnosis and treatment. In our patient, a negative infection screen, negative microscopy for acid-fast bacilli and absence of haemophagocytosis on bone marrow biopsy helped exclude important differentials, especially crucial given that the location of lymphadenopathy here was less typical for KFD.

It must be noted that the association between KFD and adult-onset Still’s disease (AOSD) was considered, given the elevated ferritin and the triggering infection; however, the positive ANA, lymph-node biopsy result and pancytopenia in the absence of haemophagocytosis in the bone marrow did not support this. Given initial uncertainty, high dose corticosteroids were used as treatment.

The association with infection and autoimmunity may provide insight into the aetiology of KFD, with viral infections being the most recognised triggers. Our patient developed a clinical syndrome and histology consistent with KFD following bacterial cellulitis. His development of autoimmune hypothyroidism and the subsequent SLE-like syndrome highlights the association between KFD and autoimmunity. The evolution of SLE-like disease was predicted by the positive ANA, however, it is interesting to note that his initial anti-dsDNA titre and complement levels were normal. The development of dsDNA antibodies and a low C4 implies SLE-type pathology, described in the literature to be associated with KFD. This serological change was responsive to hydroxychloroquine, suggesting ongoing pathology, years following the initial presentation. The hydroxychloroquine was continued as cessation caused anaemia and recurrence of this SLE-type serology. As a point of discussion, prolonged rheumatology follow up in KFD may be useful as pathology may persist for some time after the initial clinical presentation.

**Key learning points/Conclusion:**

Kikuchi-Fujimoto disease is a rare but recognised cause of pyrexia and lymphadenopathy; it should be considered as a differential in the appropriate clinical scenario. The authors suggest that the rheumatologist should play a key role in ensuring that this diagnosis is considered when consulting with colleagues as part of multidisciplinary team discussions.

Kikuchi-Fujimoto disease has overlap with signs and symptoms of SLE when occurring independently but may be associated with co-existent rheumatic disease, including SLE or Still’s disease.

Infections from both bacterial and viral pathogens can potentially trigger Kikuchi-Fujimoto disease.

It is important to perform a lymph node biopsy and exclude differentials such as occult infections, tuberculosis and lymphoma.

Where haemophagocytic lymphohistiocytosis is suspected, bone marrow trephine is also mandated.

High dose corticosteroids can be used to successfully treat severe clinical manifestations of Kikuchi-Fujimoto disease.

Recurrence of pathology may occur late in the disease and patients should remain under the surveillance of a rheumatologist for several years after initial presentation, especially where initial illness is severe or ANA is positive.

